# Supplementary material for: Comparative genomics provides new insights into the diversity, physiology, and sexuality of the only industrially exploited tremellomycete: Phaffia rhodozyma
Source: BMC Genomics. 2016 Nov 9;17:901. doi: 10.1186/s12864-016-3244-7 (PMC5103461; doi:10.1186/s12864-016-3244-7)
Supplement: Additional file 6: — List of orphan genes with links to PFAM (related to Additional file 1: Table S1). (ZIP 1428 kb) [file 12864_2016_3244_MOESM6_ESM.zip › PFAM_UNIQUE/G03814_P.html]

#                                                                            --- full sequence --- -------------- this domain -------------   hmm coord   ali coord   env coord
  
# target name        accession   tlen query name           accession   qlen   E-value  score  bias   #  of  c-Evalue  i-Evalue  score  bias  from    to  from    to  from    to  acc description of target
  
#------------------- ---------- ----- -------------------- ---------- ----- --------- ------ ----- --- --- --------- --------- ------ ----- ----- ----- ----- ----- ----- ----- ---- ---------------------
  
G03814\_P             -            495 DUF1180              PF06679.7    163      0.63    8.6  14.0   1   1     0.019        23    3.5  14.0    38   122    67   192    31   196 0.58 - Protein of unknown function (DUF1180)  
G03814\_P             -            495 DUF1970              PF09301.5    117       4.7    6.0   8.3   1   2       1.1   3.4e+03   -3.3   4.5    47    85    61    98    37   126 0.69 - Domain of unknown function (DUF1970)  
G03814\_P             -            495 DUF1970              PF09301.5    117       4.7    6.0   8.3   2   2   9.5e-05      0.28    9.9   0.1    36   109   367   438   355   442 0.71 - Domain of unknown function (DUF1970)  
G03814\_P             -            495 DUF3328              PF11807.3    217       4.9    5.4  12.3   1   3      0.02        30    2.8   2.7     3    69    81   145    34   152 0.74 - Domain of unknown function (DUF3328)  
G03814\_P             -            495 DUF3328              PF11807.3    217       4.9    5.4  12.3   2   3   0.00049      0.73    8.1   0.0     3    79   156   249   154   270 0.57 - Domain of unknown function (DUF3328)  
G03814\_P             -            495 DUF3328              PF11807.3    217       4.9    5.4  12.3   3   3      0.26   3.9e+02   -0.8   0.6    27    85   384   443   377   463 0.69 - Domain of unknown function (DUF3328)  
G03814\_P             -            495 DUF4448              PF14610.1    189     0.016   13.4   0.1   1   2   5.7e-05     0.038   12.1   0.0   155   186   159   192    96   194 0.70 - Protein of unknown function (DUF4448)  
G03814\_P             -            495 DUF4448              PF14610.1    189     0.016   13.4   0.1   2   2       5.9   3.9e+03   -4.2   0.1    21    30   401   410   395   412 0.74 - Protein of unknown function (DUF4448)  
G03814\_P             -            495 DUF912               PF06024.7    101     0.018   13.8   0.4   1   2     4e-05      0.04   12.6   0.0    60    95   161   195   109   203 0.75 - Nucleopolyhedrovirus protein of unknown function (DUF912)  
G03814\_P             -            495 DUF912               PF06024.7    101     0.018   13.8   0.4   2   2       3.3   3.3e+03   -3.1   0.5    31    45   347   361   335   382 0.46 - Nucleopolyhedrovirus protein of unknown function (DUF912)  
G03814\_P             -            495 Glycophorin\_A        PF01102.13   122   4.4e-05   21.9   0.6   1   3       1.1   3.2e+03   -3.5   3.7    14    30    49    68    28   101 0.51 - Glycophorin A  
G03814\_P             -            495 Glycophorin\_A        PF01102.13   122   4.4e-05   21.9   0.6   2   3   1.5e-08   4.4e-05   21.9   0.6     6   101   108   205    95   220 0.72 - Glycophorin A  
G03814\_P             -            495 Glycophorin\_A        PF01102.13   122   4.4e-05   21.9   0.6   3   3      0.82   2.5e+03   -3.1   0.4    33    33   410   410   377   441 0.50 - Glycophorin A  
G03814\_P             -            495 Rax2                 PF12768.2    281    0.0023   15.9   0.1   1   1   2.3e-06    0.0046   14.9   0.1   196   261   122   197    95   207 0.71 - Cortical protein marker for cell polarity  
G03814\_P             -            495 SLAC1                PF03595.12   330     0.043   11.1   0.0   1   1   6.6e-05     0.056   10.7   0.0   103   208    93   206    26   208 0.76 - Voltage-dependent anion channel  
G03814\_P             -            495 SOBP                 PF15279.1    306         1    8.3  14.2   1   2    0.0016      0.22   10.5   4.5    68   145    51   129    24   282 0.71 - Sine oculis-binding protein  
G03814\_P             -            495 SOBP                 PF15279.1    306         1    8.3  14.2   2   2      0.29        40    3.1   2.2   101   217   393   455   318   488 0.34 - Sine oculis-binding protein  
G03814\_P             -            495 TMEM154              PF15102.1    146     0.019   13.3   2.4   1   1   0.00018      0.11   10.9   2.4    19    97   112   200    20   216 0.64 - TMEM154 protein family
